# Dr. Negrier on a Very Simple Means of Arresting Epistaxis

**Published:** 1842-11-05

**Authors:** 

**Affiliations:** Angers


*On a very simple means of arresting Epistaxis.* By Dr. Negrier, of Angers.—This consists in nothing more than closing with the opposite hand the nostril from which the blood flows, while the arm of the same side is raised perpendicularly above the head. In every instance in which he has had recourse to this means during the past three years, M. Négrier has always found that it suspended the hemorrhage: a fact of which he offers the following explanation.

When a person stands in the ordinary posture, with his arms hanging down, the force needed to propel the blood through his upper extremities is about half that which would be required if his arms were raised perpendicularly above his head. But since the force which sends the blood through the carotid arteries is the same as that which causes it to circulate through the brachial arteries, and there is nothing in the mere position of the arms above the head to stimulate the heart to increased action, it is evident that a less vigorous circulation through the carotids must result from the increased force required to carry on the circulation through the upper extremities.—*Ibid, from Archives Géneralés de Médecine.* June, 1842.

